# Spatial autocorrelation in uptake of antenatal care and relationship to individual, household and village-level factors: results from a community-based survey of pregnant women in six districts in western Kenya

**DOI:** 10.1186/1476-072X-12-55

**Published:** 2013-12-07

**Authors:** Wendy Prudhomme O’Meara, Alyssa Platt, Violet Naanyu, Donald Cole, Samson Ndege

**Affiliations:** 1 School of Medicine, Duke University, Durham, U.S.A; 2 Duke Global Health Institute, Durham, U.S.A; 3 School of Public Health, Moi University College of Health Sciences, Eldoret, Kenya; 4 School of Medicine, Moi University College of Health Sciences, Eldoret, Kenya; 5 Division of Global Health, Dalla Lana School of Medicine, University of Toronto, Toronto, ON, Canada

## Abstract

**Background:**

The majority of maternal deaths, stillbirths, and neonatal deaths are concentrated in a few countries, many of which have weak health systems, poor access to health services, and low coverage of key health interventions. Early and consistent antenatal care (ANC) attendance could significantly reduce maternal and neonatal morbidity and mortality. Despite this, most Kenyan mothers initiate ANC care late in pregnancy and attend fewer than the recommended visits.

**Methods:**

We used survey data from 6,200 pregnant women across six districts in western Kenya to understand demand-side factors related to use of ANC. Bayesian multi-level models were developed to explore the relative importance of individual, household and village-level factors in relation to ANC use.

**Results:**

There is significant spatial autocorrelation of ANC attendance in three of the six districts and considerable heterogeneity in factors related to ANC use between districts. Working outside the home limited ANC attendance. Maternal age, the number of small children in the household, and ownership of livestock were important in some districts, but not all. Village proportions of pregnancy in women of child-bearing age was significantly correlated to ANC use in three of the six districts. Geographic distance to health facilities and the type of nearest facility was not correlated with ANC use. After incorporating individual, household and village-level covariates, no residual spatial autocorrelation remained in the outcome.

**Conclusions:**

ANC attendance was consistently low across all the districts, but factors related to poor attendance varied. This heterogeneity is expected for an outcome that is highly influenced by socio-cultural values and local context. Interventions to improve use of ANC must be tailored to local context and should include explicit approaches to reach women who work outside the home.

## Background

Globally, nearly 300,000 thousand women die each year as a result of pregnancy related complications [1], 2.6 million babies are estimated to be stillborn [2], and 4 million more newborns die in the neonatal period [3]. Many of these deaths could be prevented through interventions delivered as part of basic antenatal care services such as micronutrient supplementation and nutrition education, malaria prevention, tetanus toxoid immunization, HIV and syphilis screening, and screening and treatment of pre-eclampsia and other hypertensive disorders [4-8]. Mothers who attend ANC, and specifically those who receive counseling on birth preparedness, may be more likely to deliver with a skilled birth attendant [9,10].

Overall, prevention interventions delivered during antenatal care can address a large fraction of the causes of neonatal deaths. Despite the potential for focused antenatal care initiated early in pregnancy to save lives and improve the health of both the mother and the baby, only 85.8% of Kenyan women received any antenatal care from a skilled professional during their last pregnancy [11]. Even more alarming, 40% attended their *first* ANC visit after 6 months gestation and less than half of women attended the recommended minimum 4 visits [12]. Late attendance at ANC limits the delivery and effectiveness of interventions such as tetanus immunization, screening for anaemia, pre-eclampsia and gestational diabetes, nutrition supplementation, antiretrovirals for prevention of vertical HIV transmission, and intermittent preventive therapy for malaria in pregnancy (IPTp).

In Kenya, basic antenatal services are delivered predominantly by nurse-midwifes at all levels of the health system, from peripheral health facilities (dispensaries, health centres) all the way up to district and referral hospitals. The 2010 Kenya Service Provision Assessment survey showed that 88% of all health facilities offer basic antenatal care and at least 80% of health centres offer tetanus immunization and routine syphilis screening in ANC. In addition, of those facilities that do offer ANC, 75% do so 5 or more days per week [13]. Given the availability of services in the health sector, it is important to understand the demand side factors that contribute to poor uptake of antenatal services. These factors are multifaceted and complex, including physical and financial accessibility, perceived value of services, perceived or actual poor quality, mother’s education or understanding of the benefits of ANC, cultural beliefs about pregnancy, influence of other family members on care seeking patterns, and the tension between traditional birth practices and modern medicine [10,14-21].

Here we compare ANC uptake across six districts in western Kenya with differing access to care, socioeconomic conditions, and cultures. We evaluate the relative importance of individual, household and community factors in determining whether or not a woman attends antenatal care. In particular, we look at whether we can identify subpopulations of pregnant women who have low ANC uptake either geographically or demographically and discuss how our results may inform interventions to reach these women.

## Methods

### Study districts

**Figure 1:**
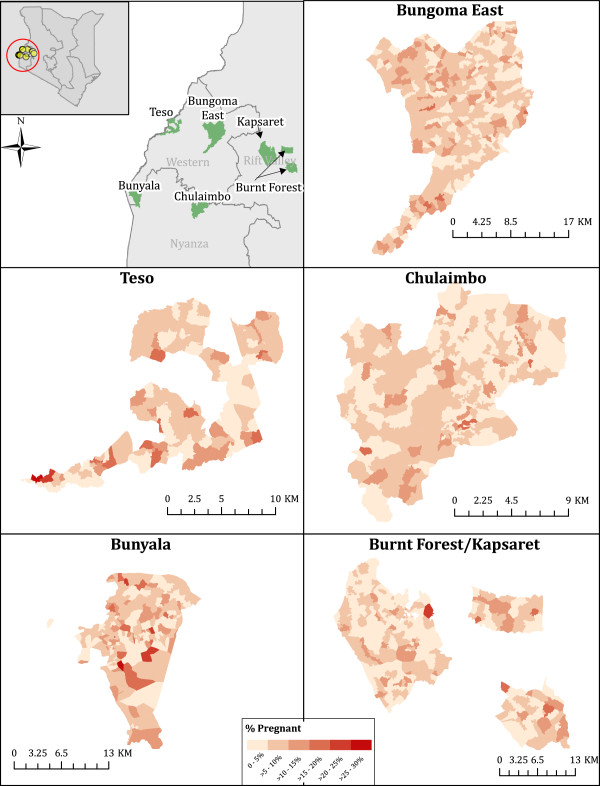
Percentage of women (age 13-45) who report being pregnant, by village.

The six districts included in this analysis – Bungoma East, Burnt Forest, Kapsaret, Bunyala, Chulaimbo, and Teso North – are distributed across western Kenya (Figure 1) but are all part of the catchment area of the Academic Model Providing Access to Healthcare (AMPATH). Each district has a district hospital and between 7 to 11 peripheral health facilities within the district. The districts vary in ethnic composition, population density, economic activities, access to health services, and disease burden. Burnt Forest and Kapsaret are more urban than the other districts and are predominantly settled by the Kalenjin people, although the town centers are more diverse. Malaria transmission is low to absent. Chulaimbo is populated mostly by the Luo ethnic group. Bunyala lies on the shores of Lake Victoria and the main economic activity is fishing. Most people belong to the Luhya ethnic group with a smaller number of Luo families. Malaria transmission in Bunyala and Chulaimbo is intense and perennial and the HIV burden is also very high. In Bungoma East, sugar cane is an important cash crop and most families engage in cash crop farming as well as subsistence farming and animal husbandry. The major ethnic group is the Bukusu which is a subtribe of the Luyha. Teso North district borders Uganda and is inhabited by the Teso people. Malaria transmission in Teso North and Bungoma East is also very high and transmission is experienced year round. Unlike Chulaimbo and Bunyala, the HIV burden is much lower.

### Study population

Pregnant women were identified during a large home-based HIV counseling and testing program (HBCT) in six districts in western Kenya conducted between 2009–2011. Women of reproductive age were asked whether they are currently pregnant and if so, whether they had received antenatal care for their current pregnancy. Our sample consists of women over the age of 13 years in each district who self-reported their pregnancy in the survey. At each stage of our analysis, the population of pregnant women is always the denominator.

### Data collection

Details of the HBCT program and data collection are presented in detail elsewhere [22]. As part of the survey, basic demographic information, GPS coordinates, and socioeconomic information were collected at each household. Individual information such as age, marital status, and working hours were recorded for those who consented to counseling.

### Ethical approval

HBCT is a home-based public health initiative. All participants gave voluntary informed consent for HIV testing. Consent was obtained verbally prior to data collection or any test being conducted. In the context of a community health initiative, written consent was not considered appropriate. Verbal consent is considered the norm for most clinical care procedures and activities in our region. Documentation of verbal informed consent was collected by recording who had accepted household entry and testing.

The Institutional Review and Ethics Committee at Moi University and Moi Teaching and Referral Hospital in Eldoret, Kenya and Duke University Institutional Review Board approved the use of de-identified data from this program for analysis and publication.

### Spatial variables

Spatially derived variables associated with geographic access to ANC care were: calculated distance to the nearest national road (Africover; last updated 2003), distance to the nearest health facility, and the type of facility nearest a woman’s household. Proximity to national roads and health facilities could conceivably have a non-linear relationship with use of antenatal care, therefore we also tested the use of a quadratic distance term as well as a log distance term as alternatives to linear distance, however, the non-linear terms did not improve model fit so a linear term was used. For five districts, distances were calculated as Euclidean distance which was necessitated by a lack of geographic data on local roads and footpaths, the most likely route of travel to a health facility. Although not a precise measure of travel distance, straight-line distance still provides an acceptable proxy for a woman’s proximity to resources for ANC care. In Bunyala, spatially derived variables were more complex. This district is bisected by two main rivers and several areas of the district are located in close proximity to wetland areas likely to limit a woman’s access to care. Based on knowledge of local geography, we assumed that women would not access care by crossing either of the two main rivers going south towards the more rural part of the district, though they may cross the rivers travelling north towards the hospital and main town center. Furthermore, when a river needs to be crossed, an individual must use an established crossing (bridge or ferry point) to safely traverse the river in order to reach a road or a health facility. Thus when the closest road or facility was located across a river to the north, the distance was calculated first from the household to the river crossing, then from the river crossing to the point of interest. If this distance was still the shortest distance to a road or facility, then this was the distance used for the derived variable of interest.

The survey included location information such as broad administrative district, location, sublocation, and village, in addition to household GPS coordinates. Because the village unit is considered an important geographic as well as cultural unit, and maps of village boundaries are not available, village boundary maps were constructed by the study team using households’ GPS coordinates and village affiliations listed in the survey. Thiessen polygons were constructed from the household coordinates and then dissolved by village and clipped using the administrative boundaries of each district. Due to error in some GPS coordinates and use of village cultural association in place of geographic village location, village shapefiles were inspected and edited by hand where errors were apparent. All geographic data manipulation was conducted in ArcGIS 10.

### Local clustering

We conducted a preliminary exploratory spatial analysis by testing the population at risk of ANC non-attendance (in our case the population of pregnant women) in each district for autocorrelation in ANC attendance. Since our outcome is binary, we chose to run a join count test [23,24]. Our version of the join count looks at all pairs of neighboring points (locations at which a pregnant woman resides). The neighboring points will be one of three possible combinations: two pregnant women who both attend ANC, one pregnant woman who attends ANC and one who does not, and two pregnant women who do not attend ANC. Because we are interested in autocorrelation of ANC attendance, the pairs are designated as positive “joins” if they are of the same type (in our case, a pair of women who attended ANC). The test statistic is computed by taking the difference between the expected number of joins (given the total number of pairs of neighboring women and the total ratio of women attending ANC versus not) and the actual number of joins and determining whether the difference deviates significantly from a pattern that would be expected under spatial randomness. A higher than expected number of joins of the same type suggests positive spatial autocorrelation. A weights matrix is required which defines whether a relationship exists between two observations, in other words whether they are defined as ‘neighbors’. Neighborhoods (or definitions of “neighbor”) can be defined by proximity, e.g. some number of nearest neighbors to an observation or a distance band, which defines a set radius around an observation and considers every observation within the area to be a “neighbor.” In computation of autocorrelation statistics such as the join count, it is common to specify multiple levels and types of weight matrices [23]. We defined neighborhood in 4 ways; 8 nearest neighbors, 10 nearest neighbors, the maximum distance to the nearest neighbor, and 1.5 times the maximum distance to nearest neighbor. We used the spdep package in R 3.0 [25] to compute the join count test statistic and p-value which tests only for positive autocorrelation.

### Multivariable hierarchical models

Our final sample for regression analysis was composed of the population of pregnant women in each district. Bayesian models were specified at each level with and without uncorrelated heterogeneity (an independently distributed random effect) and with or without correlated heterogeneity (spatial autocorrelation term, using Besag’s model). The spatial random effect, via Besag’s model [26,27] defines a spatially structured residual, based on contiguity of village areas, using an intrinsic conditional autoregressive (iCAR) structure. Spatial random effects were specified with binary weights matrices based on queen contiguity (two villages are neighbors if they share at least one point on their borders) of village polygons. The uncorrelated effect adds an unstructured residual with exchangeable correlation between villages. For ease of interpretation of village level heterogeneity, we calculated median odds ratios (MOR) for the random effects of each of the districts. MORs can be interpreted as the odds ratio for the difference in likelihood of adherence between two persons with the same covariates, one from a village of higher adherence and one from a village of lower adherence [28]. The MOR is a more commonly used measure of variation between random effects in regressions with binary outcome variables [29,30] as it provides a more intuitive interpretation than the variance of the random effects.

Logistic regression models were estimated using the Integrated Nested Laplace Approximation (INLA) package in R 3.0 [31]. INLA is a relatively new method used to estimate latent Gaussian Markov Random Fields and is a computationally efficient alternative to MCMC methods for estimating Bayesian models including those with spatial heterogeneity [32].

Models were compared using the Deviance Information Criterion (DIC) and Moran’s I test of the deviance residuals. DIC considers the posterior mean deviance and adjusts for model complexity via the numbers of effective parameters. DIC is often used to compare and evaluate the fit of variations of the same regression model in Bayesian analysis and smaller scores are preferred [33]. We used Moran’s I test for autocorrelation of the deviance residuals to determine whether additional spatial autocorrelation remained after adjusting for covariates or adding village-level uncorrelated heterogeneity effects. We also compared these results to Moran’s I test of the deviance residuals of the unadjusted model [34,35].

## Results

### Study population

99,915 women of reproductive age (13 to 45 years old) were interviewed across the six districts. Overall, 6.2% reported being pregnant although the proportion pregnant by village varied from 0 up to 30%. Amongst pregnant women, 59.4% said they had attended ANC care for their current pregnancy. Reported ANC attendance varied between the districts from 48-68% (Table 1).

**Table 1:** Descriptive statistics by district

	**Bungoma East**	**Teso North**	**Chulaimbo**	**Bunyala**	**Burnt forest**	**Kapsaret**	
	***N***	***N***	***N***	***N***	***N***	***N***	
Total population	187665	44,301	52,674	56,670	63,472	56,006	
Total households	46,826	11,470	15,663	17,443	18,015	17,706	
Total women age 13 to 45 (interviewed)	43,739	7,083	13,060	13,958	6,988	15,087	
Pregnant women (analysis samp.)	3008	521	736	1031	299	582	
**Variables**	**Mean ± SD**	**Mean ± SD**	**Mean ± SD**	**Mean ± SD**	**Mean ± SD**	**Mean ± SD**	P-value*
**Outcome variable**							
Attended ANC (%)	0.570	0.678	0.683	0.574	0.475	0.636	
**Individual level**							
Age (%)							*0.535*
Age < 18	**5.8**	**3.3**	**8.7**	**3.7**	**2.3**	**1.4**	
Age 18 to 24	**45.1**	**46.1**	**46.5**	**53.3**	**34.8**	**39.2**	
Age 25 to 34	**37.3**	**39.9**	**38.7**	**35.4**	**47.2**	**47.9**	
Age 35+	**11.8**	**10.7**	**6.1**	**7.6**	**15.7**	**11.5**	
Marital status (%)							*<0.001*
Married/Partnered	**88**	**82.5**	**74.6**	**73.2**	**75.9**	**77**	
Divorced/Separated/Widowed	**2.8**	**3.1**	**6**	**6.1**	**3.7**	**4.1**	
Never married	**9.2**	**14.4**	**19.4**	**20.7**	**20.4**	**18.9**	
Housework (%)							*<0.001*
No housework	**6.9**	**5.4**	**4.9**	**3.1**	**6**	**4.5**	
10 hours or less/week	**47.5**	**18**	**17.1**	**21.3**	**21.4**	**12.5**	
11 to 34 hours/week	**24.6**	**39.9**	**48.8**	**41**	**43.1**	**47.3**	
Greater than 35 hours/week	**21.1**	**36.7**	**29.2**	**34.5**	**29.4**	**35.7**	
Work for pay (%)							*<0.001*
No work for pay	**76.8**	**65.5**	**64.9**	**62.9**	**47.8**	**58.9**	
10 hours or less/week	**8.8**	**8.3**	**9**	**8.1**	**13**	**8.6**	
11 to 34 hours/week	8.8	15.7	17.4	19.8	22.4	17.4	
Greater than 35 hours/week	5.5	10.6	8.7	9.3	16.7	15.1	
**Household Level**	Mean ± SD	Mean ± SD	Mean ± SD	Mean ± SD	Mean ± SD	Mean ± SD	
Total household members	**4.19** ± 2.17	**3.97** ± 2.06	**3.80** + 1.76	**3.65** ± 1.86	**4.09** ± 2.05	**3.43** ± 1.68	*<0.001*
Number of children ≤5y in HH	**1.074** ± 0.927	**0.887** ± 0.768	**1.076** ± 0.865	**0.887** ± 0.778	**0.779** ± 0.713	**0.631** ± 0.645	*<0.001*
Number of children 6 –13y in HH	**0.915** ± 1.225	**1.031** ± 1.234	**0.913** ± 1.145	**0.884** ± 1.159	**1.127** ± 1.230	**0.923** ± 1.096	*0.0145*
Nearest health facility (%)							*0.0002*
Dispensary	**64.6**	**48.4**	**59.8**	**54**	**39.8**	**58.8**	
Health center	**18.7**	**47.2**	**16**	**14.6**	**60.2**	**41.2**	
Hospital	**16.7**	**24.2**	**24.2**	**31.3**			
Distance to nearest health facility (meters)	**2157** ± 1020	**2476** ± 1430	**1759** ± 844	**1867** ± 1048	**3438** ± 1512	**2498** ± 1413	*<0.001*
Distance to road (meters)	**2049** ± 1546	**3826** ± 2109	**1717** ± 1212	**1415** ± 1549	**3015** ± 2005	**2443** ± 1958	*<0.001*
Total animals owned by household	**1.300** ± 2.057	**1.468** ± 2.303	**1.531** ± 2.808	**1.051** ± 2.495	**3.652** ± 6.437	**2.007** ± 3.257	*<0.001*
Household owns land (%)	**75.5**	**74.3**	**64.3**	**51.9**	**57.9**	**69.2**	*<0.001*
**Village level**							
Fraction of children attending school (%)	**79.8**	**83.5**	**85.3**	**79**	**86.1**	**85.2**	*<0.001*
Average number of animals per HH	**1.687** ± 0.525	**1.752** ± 0.702	**2.155** ± 0.767	**1.051** ± 2.495	**4.378** ± 1.596	**2.094** ± 0.503	*<0.001*
Fraction of households owning land (%)	**75.4**	**77.5**	**71.1**	**57.2**	**60.4**	**69.9**	*<0.001*
Pregnancies in village (per 1000 WRA)	**87.2** ± 32.8	**99.3** ± 54.1	**73.7** ± 32.0	**96.2** ± 50.6	**91.6** ± 38.2	**71.6** ± 33.7	*<0.001*

*Differences between districts compared using one-way ANOVA with Bonferroni corrections for continuous variables and Cochran–Mantel–Haenszel statistics for binary/categorical variables.

Pregnancy rates across most of the villages in the 6 districts appear to exhibit a spatially random pattern (Figure 1). To confirm that village proportions of pregnant women were in fact random, we performed a Global Moran’s I test for spatial autocorrelation revealing positive autocorrelation (p = 0.023) in Teso North, but no significant autocorrelation in any of the other districts. This suggests that proportions of women of reproductive age that are pregnant do not tend be concentrated in specific geographic areas.

Most pregnant women were married or partnered. 31.3% reported working for some hours each week outside the home. 30.7% reported hours spent each week doing both housework and work outside the home. Generally, the hours worked outside the home were fewer than the hours spent on housework. Burnt Forest had the largest proportion of women working outside the home. The hours a pregnant woman spent working outside the home in Bungoma East was half to a third of other districts.

The average household size was 3.4 to 4.2 members and differed significantly across districts. Within the household, there was on average one child under 5 years and one child between the ages of 6–13 years. Land ownership was highest in Bungoma East, where three-quarters of households owned land, and lowest in Bunyala where only 50% of households owned land. 28-63% of households owned livestock such as chickens, goats, sheep and cows.

The average Euclidean distance between a household and the nearest government health facility was between 1.8 km and 3.4 km with the largest distances in Burnt Forest and the smallest in Chulaimbo. The nearest major road was on average 0.5 km nearer than the nearest health facility in three districts, and roughly equidistant in the remaining three districts.

School attendance was high across the districts. The village average of children ages 3–20 years attending school was between 78-86%. The mean proportion of women pregnant was highest in Bunyala (96.2 per 1000 women of reproductive age).

### Geographic autocorrelation of women not attending ANC

It was hypothesized that use of ANC may be influenced by local factors including physical access to clinics offering ANC, family assets, and other unmeasured factors related to family or community beliefs and values about health care, pregnancy or antenatal care. All of these factors could lead to geographic autocorrelation of women attending or not attending ANC services.

Results of the analysis of spatial autocorrelation in ANC attendance evaluated using the join count statistic are presented in Table 2. We found significant spatial autocorrelation in ANC attendance in Bungoma East, Chulaimbo and Kapsaret for at least one definition of neighborhood.

**Table 2:** Join count test: P-values

	**Neighborhood definition**			
**District**	**8 NN**	**10 NN**	**Max distance**	1.5* Max distance
Bungoma East	**0.035**	0.153	**0.006**	**0.000**
Teso North	0.077	0.173	0.187	0.164
Chulaimbo	**0.031**	0.137	0.330	0.836
Bunyala	0.302	0.361	0.750	0.460
Burnt Forest	0.415	0.476	0.600	0.317
Kapsaret	**0.035**	**0.007**	**0.007**	0.722

*Significant autocorrelation (p<0.05) in **bold.**

The spatial extent of the neighborhoods used to describe clustering was guided by the average area of a village. In absolute distance, a neighborhood defined as the 8 nearest neighbors ranged between 0.5 and 1 km. A neighborhood defined based on the maximum distance to the nearest neighbor ranged between 0.85 km and 1.9 km (Additional file 1: Table S1). Specifically, the radius of 8 nearest neighbors correlated closely with average village size. The neighborhoods corresponding to maximum distance to nearest neighbor and 1.5 times the maximum distance encompassed approximately two neighboring villages.

### Multi-level model of ANC uptake

The factors potentially contributing to spatial autocorrelation revealed by the join count results could occur at the individual level, the household level, or the village level. The neighborhood definitions suggested local context at the scale of the village could be important in defining clusters or subpopulations of women at risk of low attendance at ANC care. The final multi-level models of ANC attendance for each district that sequentially incorporated individual, household and village covariates are presented in Table 3.

**Table 3:** Logistic regression with village level random intercepts

	**Bungoma East (N = 3008)**			**Teso North (N = 521)**			**Chulaimbo (N = 736)**			**Bunyala (N = 1031)**			**Burnt Forest (N = 299)**			**Kapsaret (N = 582)**		
	**OR**	**(95% CI)**		**OR**	**(95% CI)**		**OR**	**(95% CI)**		**OR**	**(95% CI)**		**OR**	**(95% CI)**		**OR**	**(95% CI)**	
(Intercept)	1.977	1.213	3.233	0.815	0.220	3.022	5.980	1.862	20.60	0.880	0.339	2.312	1.460	0.338	6.461	1.173	0.367	3.874
Age (ref. Age 35+)																		
Age < 18	0.783	0.515	1.191	1.584	0.438	6.190	0.479	0.178	1.259	0.895	0.385	2.074	2.324	0.378	15.44	0.384	0.071	1.875
Age 18 to 24	1.254	0.941	1.670	2.150	0.986	4.681	0.870	0.380	1.915	**1.836**	**1.058**	**3.192**	1.370	0.578	3.291	1.237	0.623	2.440
Age 25 to 34	1.188	0.912	1.548	1.621	0.822	3.181	0.810	0.370	1.701	1.591	0.927	2.734	**2.241**	**1.033**	**4.951**	1.472	0.797	2.701
Marital Status (ref. Single/Never Married)																		
Married/Partnered	0.829	0.625	1.097	0.810	0.432	1.482	0.829	0.511	1.330	0.975	0.691	1.371	0.464	0.232	0.913	1.375	0.854	2.211
Divorced/Separated/Widowed	1.152	0.678	1.971	1.417	0.388	5.835	1.186	0.517	2.809	0.870	0.469	1.615	**0.072**	**0.010**	**0.399**	0.901	0.343	2.393
Housework (ref. No housework)																		
10 hours or less/week	**0.700**	**0.502**	**0.969**	**3.482**	**1.338**	**9.193**	0.642	0.244	1.580	1.041	0.461	2.305	0.658	0.181	2.328	1.417	0.496	3.952
11 to 34 hours/week	**0.612**	**0.431**	**0.865**	**3.437**	**1.439**	**8.266**	0.762	0.307	1.752	1.044	0.480	2.224	0.896	0.271	2.884	1.289	0.507	3.149
Greater than 35 hours/week	**0.689**	**0.483**	**0.979**	**2.945**	**1.236**	**7.075**	0.517	0.206	1.207	0.887	0.408	1.886	0.652	0.197	2.098	0.893	0.345	2.215
Work for pay (ref. No work for pay)																		
10 hours or less/week	0.866	0.658	1.139	**0.378**	**0.171**	**0.839**	0.656	0.363	1.196	1.508	0.875	2.641	0.889	0.346	2.269	0.906	0.438	1.905
11 to 34 hours/week	0.768	0.585	1.009	0.621	0.354	1.098	1.294	0.810	2.096	**0.701**	**0.497**	**0.987**	1.028	0.513	2.059	0.759	0.456	1.265
Greater than 35 hours/week	0.723	0.516	1.013	0.657	0.344	1.270	0.731	0.412	1.311	**0.617**	**0.387**	**0.982**	**0.378**	**0.173**	**0.804**	0.689	0.407	1.170
Total household members	0.926	0.667	1.286	0.881	0.384	2.026	1.359	0.626	2.995	1.072	0.593	1.949	1.041	0.346	3.177	1.033	0.496	2.175
Number of children ≤5y in HH	0.841	0.681	1.038	0.968	0.590	1.589	**0.595**	**0.358**	**0.979**	**0.601**	**0.417**	**0.861**	0.896	0.468	1.710	0.828	0.527	1.299
Number of children 6 –13y in HH	0.979	0.748	1.281	1.020	0.489	2.127	0.857	0.427	1.715	1.106	0.658	1.854	1.296	0.490	3.442	1.106	0.543	2.250
Nearest health facility (ref. Dispensary)																		
Health center	1.090	0.849	1.399	0.857	0.521	1.407	0.990	0.588	1.673	1.191	0.764	1.863	0.814	0.412	1.608	0.948	0.566	1.576
Hospital	1.052	0.822	1.347	0.310	**0.108**	**0.891**	0.749	0.483	1.157	1.024	0.707	1.485						
Distance to nearest health facility	0.948	0.796	1.130	1.013	0.597	1.730	0.838	0.582	1.202	0.931	0.695	1.245	1.484	0.799	2.767	1.146	0.750	1.750
Distance to road	**1.229**	**1.014**	**1.490**	0.772	0.480	1.225	1.165	0.794	1.709	1.271	0.920	1.763	1.294	0.633	2.658	1.160	0.698	1.929
Total animals owned by household	1.132	0.961	1.339	1.127	0.738	1.754	1.129	0.783	1.682	**1.650**	**1.188**	**2.355**	1.894	0.928	4.328	0.955	0.656	1.421
Household owns land	1.037	0.847	1.270	0.848	0.493	1.448	0.921	0.625	1.355	1.185	0.877	1.603	1.496	0.857	2.626	0.993	0.658	1.496
Percent of children attending school	1.058	0.881	1.271	1.222	0.755	1.991	1.213	0.848	1.738	1.184	0.890	1.577	0.857	0.507	1.452	1.342	0.902	2.005
Average number of animals	0.871	0.701	1.081	1.575	0.895	2.820	0.869	0.575	1.313	1.277	0.916	1.788	0.987	0.557	1.750	0.789	0.489	1.266
Fraction of households owning land	1.235	0.971	1.571	1.318	0.737	2.364	0.911	0.595	1.386	0.769	0.541	1.091	0.938	0.516	1.699	0.821	0.506	1.329
Pregnancies in village (per 1000)	**0.799**	**0.666**	**0.957**	0.946	0.577	1.557	1.176	0.820	1.692	**1.516**	**1.111**	**2.119**	**0.386**	**0.220**	**0.667**	0.838	0.561	1.247
Median odds ratio for village-level random effects	1.847	1.668	2.030	1.700	1.249	2.221	1.796	1.377	2.257	1.675	1.318	2.047	1.401	1.203	1.700	1.935	1.511	2.405
DIC	4076			670			947			1419			421			800		

Odds ratios reflect the mean of the posterior distribution for each parameter estimate.

Results significant at 95% level in **bold.**

Adding a village-level uncorrelated effect improved model fit in most districts (reduction of 5–32 in DIC). Addition of a correlated effect did not improve the model fit for any district and Moran’s I test of the deviance residuals of models with a random effect only revealed no residual spatial autocorrelation in the final models indicating that 1) village-level heterogeneity is important but is not necessarily related or similar to nearby villages (i.e. no spatial autocorrelation at the village-level) and 2) the included covariates adequately explain the spatial autocorrelation in ANC use that was measured by the join count statistic. Results for models with an uncorrelated random effect at the village level are therefore reported.

### Individual-level factors

In Bunyala and Burnt Forest, women between the ages of 18–34 were more likely to attend ANC than older women. In Teso North, Bunyala and Burnt Forest, women who worked outside the home for wages were 40-60% less likely to report having attended ANC. The effect of reported housework was mixed; women in Teso North who reported 10 or more hours of housework per week were at least 3 times more likely to have attended ANC but women in Bungoma East who reported 10 or more hours of housework per week were 30-40% less likely to have attended ANC. Divorced or widowed women were far less likely than single women to attend ANC in Burnt Forest (OR = 0.07, 95% CI: 0.01-0.40).

These individual factors identify groups of women at elevated risk of poor ANC uptake. For example, in Burnt Forest, women over the age of 35 who work full-time outside the home were 5.93 times less likely to receive adequate ANC care than women age 25–34 that do no work outside the home; amongst women in this category, those who are divorced, separated or widowed were 82.6 times less likely to have attended ANC care than their single counterparts. In Bunyala, older women working outside the home for more than 35 hours per week were 2.98 times less likely to attend ANC care than younger women who did not work outside the home.

### Household-level factors

The inclusion of household-level factors did not change the contribution of individual-level factors. Distance to a health facility was not correlated with ANC use in any of the districts, though increasing distance to the nearest national road was associated with an increased probability of attending ANC in Bungoma East. In Teso North, women for whom the nearest facility is a hospital were significantly less likely to have attended ANC.

In Bunyala and Chulaimbo, the odds of attending ANC was reduced by 40-60% for each additional child under 5 years of age in the household. This relationship was not seen for older children (6–13 years). More animals owned by a household were associated with an increase in the odds of attending ANC in Bunyala, though not in any of the other districts.

### Village level factors

Higher village pregnancy rates were correlated with lower ANC use in Burnt Forest and Bungoma East, but the opposite trend was observed in Bunyala where lower village pregnancy rates correlated with higher ANC use. Other village level factors were not correlated with ANC use.

Based on the median odds ratios (MOR) for the random effects, village association appears to be important in all districts, though particularly so in Bungoma East and Kapsaret where two randomly selected women with the same covariates could be between 1.85 to 1.94 times as likely to attend ANC if living in a village of higher attendance than those of lower attendance.

## Discussion

Our analysis of nearly 6,200 pregnant women across six districts showed low uptake of ANC care amongst women self-reported to be pregnant. Significant local autocorrelation of ANC attendance was apparent in three of the districts. However, geographic autocorrelation was not related to proximity to health services. Multi-level analysis revealed several factors that correlated with uptake of antenatal care, but these factors differed across the districts.

Thirty to forty percent of women had not yet attended ANC for their pregnancy. Since it is common for women to report their pregnancy only after ~20 weeks in this context, we interpret our data as a cross-sectional view of ANC attendance amongst women in their late second and third trimester. Our results agree with other studies that show that most women initiate ANC care very late in their pregnancy. Although a high percentage makes at least one visit, far fewer make two or more visits [36]. A study from western Kenya showed that 87% of women make their first ANC visit in the 2^nd^ or 3^rd^ trimester, at a mean gestational age of 5.5 months [9,15,37]. Other studies from east Africa have reported similar findings [9,37,38].

Late initiation of antenatal care is a missed opportunity and it is critical to understand the factors related to poor ANC attendance. In some studies, early ANC attendance and increasing number of visits are associated with reduced odds of premature delivery, stillbirth and perinatal death [39,40]. Using a hierarchical approach, we explored the relative importance of individual, household and village characteristics. In our models, individual, household and village level factors are all correlated to ANC use, but their importance varied between communities. In Burnt Forest, characteristics of the mother were the most significantly correlated with ANC attendance. Divorced or separated women were twice as likely to have delayed initiating ANC compared to single women. Marital status has also been correlated to early ANC use in other studies [10,20]. In three districts, working for wages was consistently negatively associated with ANC use, consistent with another western Kenyan study where engagement in more income-generating activities reduced attendance [38], indicating that time constraints may limit the opportunity to attend ANC. This is in contrast to a study using data from the country-wide 1993 Kenya Demographic and Health Survey [20] which showed that women employed outside the home were more likely to attend ANC early. In Bungoma East, increasing hours of housework reduced ANC attendance but in Teso North, women who work in the home were more likely to have attended ANC.

In Chulaimbo, individual mother characteristics were not associated with ANC attendance, but women from households with more young children were less likely to have initiated ANC care. To the extent that more young children in the home may reflect parity, this result agrees with other studies demonstrating that women experiencing their second or greater pregnancy initiate ANC care significantly later [10]. In Bunyala, women from families that owned livestock were more likely to have attended ANC. Livestock ownership is a limited proxy measure for socioeconomic status in rural, agricultural communities. Socioeconomic status has been correlated with health seeking behavior including ANC use in other studies [16,20,36] but not all [10,18].

The proportion of women of reproductive age who are pregnant in a village was the only village-level factor associated with ANC attendance. Village pregnancy was associated with low ANC attendance in Bungoma East and Burnt Forest, but the opposite was true in Bunyala. Village-level measures of wealth such as school attendance, land ownership and animal ownership were not related to ANC use.

We hypothesized that geographic proximity to a health facility or transport network (main road) would be correlated with early use of ANC services, but we did not find any evidence of this despite the heterogeneity in distance to services across the districts. Unlike other studies, we considered distance as a continuous variable objectively measured from geographic coordinates rather than using a threshold of distance or self-reported travel time. Even with this more precise measure of geographic access, our results are consistent with other studies that also failed to detect an effect of distance or travel time on ANC attendance or uptake of IPTp [36,41,42]. Similar to a countrywide analysis in Zambia [41], we did not observe significant differences in ANC uptake between mothers living closest to a dispensary as compared to a health centre or hospital except in one district. These results are in sharp contrast to what has been observed for facility delivery in Kenya and other countries (Prudhomme O’Meara W, Karuru S, Fazen L, Koech J, Kizito B, Tarus C, Menya D: *Heterogeneity in health seeking behavior for treatment, prevention and urgent care in four districts in western Kenya, Submitted*) [43,44]. Dispensaries, the most common public health facility and nearest type of facility for 43-65% of homes in our study, do not have opening hours on evenings or weekends. On average, women must travel 3–4 times as far to access a hospital with weekend operating hours. This does not however play a limiting role in mothers’ attendance at ANC.

In Bungoma East, we observed that the odds of attending ANC increased by 23% for every additional 1.5 kilometers between a household and a major road. Mothers living 5 kilometers from a major road are twice as likely to have attended ANC at the time of the survey. It is possible that women who live further from transport networks or health services are more likely to invest in travel for prevention in order to avoid the need for emergency care. It is worth noting that Brown *et al.*[18] observed a similar phenomenon on Kwale district, Kenya. In Kwale, amongst women who attended any ANC, those lived further from a health facility were more likely to initiate early and have an adequate number of visits.

The spatial autocorrelation measured by the join count statistic was accounted for by the covariates in the multi-level model. However, uncorrelated village-level random effects were still very important in optimizing model fit. Furthermore, median odds ratios revealed important village-level heterogeneity; holding all other measured covariates equal, two women living in villages with high versus low ANC attendance had nearly a two-fold difference in odds of ANC attendance. This indicates that village-level context is indispensable for explaining ANC use, but neighboring villages are not more similar to each other than non-neighboring villages. It also suggests that the village-level heterogeneity is not adequately captured by the explanatory variables in the model. Other factors that likely contribute to village-level differences in ANC use include social networks and peer influence. Women reported receiving counseling and support through church groups, traditional birth attendants, and relatives [45], all of which are likely to exert influence at the scale of a village.

There are several important limitations to consider when interpreting the results of this study. The data collected here only partially described the differences between mothers, households, and villages. In particular, mother’s education has been shown to be important in ANC use and choice of delivery location [17,18] but was not captured in this survey. Socioeconomic data is limited to land and animal ownership and school attendance. Although geographic access to health services was not correlated with ANC use, other unmeasured facility-level constraints such as quality, availability of specific services (i.e. laboratory), acceptability of services, or opening hours may play a role [14,46-48]. Our results should not be interpreted to mean that no limitations exist on the supply side of ANC care.

## Conclusions

Early and regular ANC attendance could save tens of thousands of mothers and children and reduce morbidity in infancy. Furthermore, attendance at ANC strongly predicts delivering in a health facility with a skilled birth attendant [9,10,18]. When comparing analyses of country-wide data for Kenya in 1993 [20], 2003 [10] and the current study, ANC use has changed very little in the last 20 years and the same subgroups of poor, less educated, and unmarried women are still at higher risk for low coverage of antenatal care. In our study, early ANC attendance was consistently low across all the districts, but factors related to late initiation of ANC varied. This heterogeneity is expected for an outcome that is highly influenced by socio-cultural values and local context. The only consistent observation was the lack of association between geographic access to ANC services at all levels of the health system with poor uptake of ANC. Our results suggest that interventions to improve early initiation of ANC must be tailored to local context. Approaches to improve ANC uptake should include a special focus on opportunities for women who work outside the home to access ANC services.

## Competing interests

The authors declare that they have no competing interests.

## Authors’ contribution

WPO led the conceptualization of the analysis, participated in data preparation, and drafted the manuscript. AP participated in conceptualization of the analysis, prepared and analyzed the data, and drafted the manuscript. VN participated in interpretation of results and drafting of the manuscript. DC participated in interpretation of the results and drafting of the manuscript. SN led data collection and participated in interpretation of the results. All the authors read and approved the final manuscript.

## Supplementary Material

Additional file 1: Table S1 Distance to neighbors (meters). Click here for file
