# Supplementary material for: Spatial autocorrelation in uptake of antenatal care and relationship to individual, household and village-level factors: results from a community-based survey of pregnant women in six districts in western Kenya
Source: Int J Health Geogr. 2013 Dec 7;12:55. doi: 10.1186/1476-072X-12-55 (PMC4029198; doi:10.1186/1476-072X-12-55)
Supplement: Additional file 1: Table S1 — Distance to neighbors (meters). [file 1476-072X-12-55-S1.doc]

| **Additional file 1: Table S1**  **Distance to neighbors (meters)** | | | | |
| --- | --- | --- | --- | --- |
| **District** | **Summary measure** | **Nearest Neighbor** | **8th Nearest Neighbor** | **10th Nearest Neighbor** |
| **Bungoma East** | Mean | 149.4 | 531.6 | 605.6 |
|  | Min | 0.0 | 76.5 | 126.3 |
|  | Max | 840.5 | 1487.1 | 1561.3 |
| **Teso North** | Mean | 212.1 | 807.4 | 930.2 |
|  | Min | 0.0 | 212.1 | 335.8 |
|  | Max | 1098.7 | 2531.1 | 2967.5 |
| **Chulaimbo** | Mean | 194.7 | 729.4 | 824.5 |
|  | Min | 0.0 | 230.6 | 324.1 |
|  | Max | 1031.4 | 2409.4 | 2492.3 |
| **Bunyala** | Mean | 109.2 | 484.7 | 554.6 |
|  | Min | 0.0 | 94.0 | 128.1 |
|  | Max | 1480.4 | 3236.2 | 3259.2 |
| **Burnt Forest** | Mean | 285.6 | 1130.8 | 1282.1 |
|  | Min | 0.0 | 292.6 | 314.8 |
|  | Max | 1345.3 | 2574.0 | 2701.2 |
| **Kapsaret** | Mean | 256.4 | 917.6 | 1047.0 |
|  | Min | 0.0 | 101.1 | 157.4 |
|  | Max | 1893.5 | 2566.2 | 2632.5 |
| **Mean area of villages (sq. km)** | | | | |
| **Bungoma East** | 0.914 |  |  |  |
| **Teso North** | 1.063 |  |  |  |
| **Chulaimbo** | 0.831 |  |  |  |
| **Bunyala** | 0.752 |  |  |  |
| **Burnt Forest** | 2.349 |  |  |  |
| **Kapsaret** | 1.347 |  |  |  |
